# Healthy tourism - a real need in today’s challenging society

**Published:** 2014

**Authors:** Avram Emanuela Maria, Raţiu Monica Paula

**Affiliations:** * Romanian-American University, Bucharest, Romania

**Abstract**

In recent years, healthy tourism has been attracting more consumers. Lately, many people have moved towards a healthy lifestyle and this has major implications on tourism. More and more people decided to choose destinations that can provide wellness and medical facilities. Healthy tourism became a good alternative to conventional tourism. People who want to improve their health and wellbeing choose this type of tourism. It includes: wellness tourism which satisfies the need to improve one’s health and wellbeing and the medical tourism which helps treat certain diseases at prices much lower than in the own country or take advantage of highly professional specialists and better conditions. By choosing healthy tourism, individuals benefit from relaxing, visiting new and appealing destinations, but it also has benefits for the body, mental and spiritual treatment. This paper aims to identify the particularities of healthy tourism and the impact that this type of tourism has on the increasing number of tourists. The conclusion reached from this study is that healthy tourism has become a trendy tourism, which helps promoting a healthy lifestyle with high potential of continuous growth.

**Keywords:** healthy tourism, wellness, medical tourism, lifestyle

